# Alcohol in the Early Years of Marriage

**Published:** 1996

**Authors:** Kenneth E. Leonard, Linda J. Roberts

**Affiliations:** Kenneth E. Leonard, Ph.D., is a senior research scientist with the Research Institute on Addictions, Buffalo, New York. Linda J. Roberts, Ph.D., is assistant professor in the Department of Child and Family Studies, University of Wisconsin, Madison, Wisconsin

**Keywords:** marital status, AOD use pattern, AOD consumption, marital relations, marital conflict, domestic violence, young adult

## Abstract

Marriage, a marker event for the transition from adolescence to young adulthood, brings with it many changes, including shifts in values, new roles, and adjustments in a couple’s relationship. Marriage also appears to generate shifts in alcohol use and alcohol consumption, changes that can occur even before the marriage ceremony takes place. Alcohol plays a role in marital violence, marital quality, and marital disruptions. However, high levels of individual alcohol consumption in a marriage do not uniformly lead to lower marital quality. Rather, it may be the nature of a couple’s drinking partnership (i.e., the interplay of each spouse’s drinking context and drinking patterns) that has the most effect on the health of a marriage.

One of the most important transitions in life, both generally and from an alcohol-problems perspective, is marriage. Marriage not only involves major changes but also serves as a marker event for the transition from adolescence to young adulthood. Often, but not uniformly, marriage occurs with other milestones, such as stable adult employment and parenthood. As a developmental transition, marriage carries with it a variety of tasks that can fundamentally alter both the individual’s view of him- or herself and the way in which the broader social network behaves toward the individual and the couple. Newly married people often demonstrate a marked shift away from more individualistic values and toward more interdependent and socially positive values (e.g., a greater concern for the quality of life in one’s neighborhood and a greater concern for children), consistent with the adoption of the new role of spouse. Major tasks that come with marriage involve the establishment of a mutually satisfactory relationship and the reestablishment or redefinition of ties, both as individuals and as a couple, with each member’s extended family and peer network (Boss 1983; McGoldrick and Carter 1982). Although these transitional tasks are often begun before marriage, their accomplishment continues into the early years of marriage.

Marriage also appears to engender a vital transition for newlyweds with respect to alcohol use and alcohol problems. Although much has been written on alcohol’s effect on married life, little research has taken place on the role of alcohol during the transition to and early years of marriage. This article attempts to shed some light on this important developmental time period.

## Drinking Before Marriage

Alcohol use, particularly heavy alcohol use,^1^ may come into play before the marriage itself through facilitating or delaying entry into marriage. Some evidence suggests that heavy drinking among adolescents may actually facilitate entry into marriage. For example, Power and Estaugh’s (1990) longitudinal study of British youth reported that the males who were the heaviest drinkers when surveyed at age 16 were more likely to be married at age 23 than other males. Drinking was not predictive of marriage for females. This finding was generally corroborated in a recent study by Forthofer and colleagues (1996). This effect may not be a result of the drinking per se but rather may stem from the fact that adolescents who are heavy drinkers are less likely to enter college and more likely to adopt adult roles after high school graduation. Other studies, however, have found either that drinking has no effect on selection into marriage (Bachman et al. 1984) or that alcohol problems (e.g., legal, occupational, or health problems associated with drinking) in young adulthood may delay marriage (Horwitz and White 1991). Clearly, more research is needed to clarify these contradictory findings.

Alcohol use might play a role in how people select their partners: Considerable research suggests that alcoholics are likely to be married to alcoholic spouses (see Jacob and Bremer 1986). However, this similarity could arise in four general ways: (1) selecting a partner with a similar drinking pattern or likelihood of developing alcoholism,^2^ as when a heavy drinker marries another heavy drinker (i.e., assortative mating); (2) one person influencing the other’s drinking behavior; (3) social and contextual factors that facilitate or inhibit the development of alcoholism and that operate on both members of the couple (e.g., stressful life events); and (4) couples with different drinking patterns being more likely to divorce than couples with similar drinking patterns. Thus, similarity alone does not constitute assortative mating. More indicative of assortative mating are the few studies that have examined spouses’ drinking patterns before marriage. For example, Leonard and Senchak (1993) reported that men and women who applied for a marriage license reported similar levels of alcohol use, measured in terms of average daily consumption, and alcohol problems in the year before marriage. Yamaguchi and Kandel (1993) found that husbands and wives reported similar levels of drug use before marriage. Whether these observed similarities are the result of men and women choosing marital partners of the same education, ethnicity, and social class or whether drinking or drug use plays a more direct role in the marital choice is not known.

## Effect of the Transition to Marriage on Drinking Patterns

The effect of marriage on drinking is apparent even before the marriage ceremony has been completed. For those couples who marry at a developmentally normative age (i.e., early to late twenties), researchers have observed drinking changes in the year preceding the marriage. For example, Miller-Tutzauer and colleagues (1991) used data from the National Longitudinal Survey of Youth and found that men and women who married in a given year had reduced their drinking in the year before their marriage. Bachman and colleagues (in press) replicated this effect using data from the Monitoring the Future study. This study also indicated that such an anticipatory decline only occurred among participants who reported being engaged.

These declines continue into the first year of married life. Miller-Tutzauer and colleagues (1991) observed reductions in drinking immediately after marriage, and Bachman and colleagues (in press) found that even among people who reduce their drinking during the engagement period, further declines are apparent into the first year of marriage. However, both studies found no further changes after the first year. Thus, these two studies pinpoint the transition to marriage as a critical period in the reduction of heavy drinking.

The Buffalo Newlywed Study (BNS), a 3-year prospective study of couples applying for marriage licenses, has provided a more detailed description of the changes in drinking behavior that occur during the time period preceding marriage. The BNS assessed more than 500 couples at the time they applied for their marriage license, at their first anniversary, and at their third anniversary. Couples were evaluated with respect to drinking behavior, marital characteristics (e.g., marital satisfaction, feelings of intimacy, level of conflict and quality of communication, and marital violence), and personality characteristics (e.g., hostility, masculinity, and femininity). Roberts and colleagues (1992*a*,*b*) examined the drinking behavior and contexts of drinking for the BNS couples before marriage and during the first year of marriage. They found significant declines in the frequency of alcohol consumption and in the number of alcohol problems each member of the couple experienced. These decrements could not be attributed to other developmental changes, such as getting a new job or a promotion, having a child, or simply “maturing out” of heavier drinking with advancing age.

The drinking changes that occurred in the first year of marriage were not simply a generalized reduction in average alcohol consumption; context-specific changes also took place. Although the proportion of time that the husband drank at home increased significantly over the first year, the relative amount of time he spent drinking with his wife present did not change. In addition, the typical amount he drank with his wife present declined, but the typical amount he drank in her absence did not. Thus, for men, the changes in drinking patterns related to marriage may be restricted to occasions in which the husband drinks with his wife present and to drinking in a less risky context, such as in the home. Similarly, the proportion of time that the wife drank at home also increased during the first year of marriage. Unlike the husbands, however, the wives reduced their typical quantity both when the husband was present as well as when the husband was absent. For women, then, the effect of marriage on drinking may be independent of their husband’s presence.

Despite the overall reduction in drinking and drinking problems, a sizable proportion of the married men and women did not change their alcohol consumption after marriage; some even increased their drinking during the first year of married life. Roberts and colleagues (1992*b*) reported that premarital drinking was strongly correlated with drinking patterns in the first year of marriage. For example, among men who drank four or more times per week before marriage, 44 percent reported a lower frequency for the first year of marriage. However, 56 percent of this group continued to drink at this level. Similarly, among men who drank between one and four times per month before marriage, 68 percent drank at this level or lower, but 32 percent increased their consumption. Thus, the transition to marriage leads many, but not all, couples to reduce their drinking. Identifying those who reduce their drinking and the processes responsible for this reduction will help further understanding of why some men and women continue to be heavy drinkers after marriage.

## Alcohol Effects on Early Marital Functioning

Although marriage affects drinking and drinking problems, drinking also has an effect on marital quality. It is commonly assumed that frequent or heavy drinking has a detrimental impact on marital conflict and satisfaction, but this pattern is not uniformly the case. Evidence suggests that drinking may have both positive and negative effects on a marriage.

### Alcohol and Marital Violence

One of the clearest illustrations of the potentially negative effect of alcohol on marital functioning is the commonly observed relationship between heavy drinking and domestic violence. Although much of the initial research on the subject had serious design problems, more recent research (see Leonard and Jacob 1988) has documented a clear association between heavy drinking and physical aggression in marriage. Whether and how alcohol might play a causative role in such violence, however, remains unclear and controversial. Research indicates that heavy drinking is a major risk factor for marital violence; that it *prospectively* predicts the extent of marital violence early in marriage; and that this predictive relationship cannot be attributed to sociodemographic (e.g., age), personality (e.g., hostility), or relationship (e.g., marital conflict) variables (Leonard and Senchak 1996). After controlling for premarital violence, husband’s and wife’s demographic and personality factors, and the couple’s conflict resolution styles, the husband’s alcohol use was significantly related to the extent of marital violence for BNS couples in the first year of marriage. These results corroborate the finding by Heyman and colleagues (1995) that drinking-related variables were predictive of violence early in marriage.

### Alcohol, Marital Quality, and Marital Disruptions

As might be expected, alcohol also appears to play a role in other serious marital problems. Leonard and Roberts (in press) examined the relation of married couples’ alcohol consumption and alcohol problems to declines in marital quality and to the occurrence of marital disruptions (i.e., marital separations and divorces) in the first year of marriage. Husbands’ increased alcohol consumption (measured in terms of the frequency of alcohol use and the usual quantity of alcohol consumed each day) and alcohol-related problems were related to declines in couples’ marital quality; however, these associations were not significant after controlling for personality and demographic characteristics. In contrast, husbands’ heavy alcohol consumption following marriage was uniquely predictive of marital disruptions.

The findings for wives were quite different. Neither the wives’ alcohol consumption nor their alcohol-related problems were related to marital disruption. Both variables were predictive of changes in marital quality, however: Alcohol problems were predictive of decreased marital quality, but increased alcohol consumption, after controlling for alcohol-related problems, was predictive of improved marital quality.

In cross-sectional analyses of BNS data, Roberts and colleagues (1994) again found a similar positive association: After controlling for problem drinking, wives’ and husbands’ frequent drinking predicted wives’ greater feelings of intimacy toward their husbands. This association was not evident for husbands’ feelings of intimacy toward their wives. One interpretation of these findings is that alcohol consumption not resulting in problems may actually have some positive benefits for the marital relationship. The idea that alcohol consumption may have positive effects on marital functioning should not be surprising. Research has demonstrated that although people recognize the negative behavioral effects of drinking, they also identify a number of positive effects of drinking, including increases in affective expression and enhancements in both sexual and nonsexual intimacy. For example, most women drinkers in a U.S. national survey reported that drinking made them more able to “open up” and made them feel closer to persons with whom they shared a drink (Wilsnack et al. 1987). These beliefs (i.e., expectancies), rather than the pharmacologic action of the alcohol, appear to be of critical importance (Hull and Bond 1986) with respect to several effects of alcohol, including tension reduction and sexual arousal.

## The Drinking Partnership

Our research underscores the importance of both the interpersonal context and the physical situation or setting in which the drinking takes place (i.e., the drinking context) in understanding the effects of drinking on marriage. The establishment of a shared household increases each partner’s sphere of influence over the other. Each partner becomes a salient feature of the other’s drinking context, both through direct modeling as well as through the explicit or subtle communication of attitudes and values about drinking. Marriage thus profoundly alters a person’s drinking context: With marriage, a “drinking partnership” is molded from the separate premarital drinking habits of each partner. Together, couples establish norms about such things as whether alcohol is served with meals, whether the refrigerator or alcohol cabinet is kept stocked, whether guests are offered drinks, and so on. Drinking partnerships may vary from a pattern in which the partners have one or two drinks together each night in the privacy of their home to one in which each partner separately drinks one night per week at a bar with his or her own friends. Drinking is incorporated into the fabric of a marriage in diverse ways. It may be the partnership, rather than the independent effects of one partner’s drinking, that is critical to marital functioning.

### Effect of the Drinking Partnership on Marital Quality

Analyses of the BNS data (Roberts et al. 1994) have supported this more complex conceptualization of alcohol’s effects on marriage. In addition to finding the positive association between frequent drinking and wives’ assessment of marital quality, a significant interaction existed between husbands’ and wives’ frequency of drinking (Roberts et al. 1994). Reports of higher marital satisfaction among wives were associated with drinking frequencies that were similar (i.e., *nondiscrepant*) to those of their husbands. When *both* the husband and the wife drank either frequently or infrequently, marital adjustment was higher. Nondiscrepant drinking quantities (i.e., the usual number of drinks per episode), however, were not predictive of higher marital quality for either husbands or wives.

**Table:** Drinking Partnership Profiles and Their Relationship to Marital Quality and Drinking Consequences

Drinking Partnership Type	Marital Quality	Alcohol-Related Marriage Problems^1^	General Alcohol Problems^2^
Discrepant drinkers^3^	Poor	High for the husband; low for the wife	High for the husband; low for the wife
Heavy social drinkers	Poor	Low	Moderate
Infrequent social drinkers	Good	Low	Low
Frequent at-home drinkers	Good	Low	Moderate
Infrequent at-home drinkers	Good	Low	Low

1 Alcohol-related marriage problems are marital problems that directly relate to or are exacerbated by alcohol consumption (e.g., drunken arguments).

2 General alcohol problems consist of social, legal, occupational, or health problems related to drinking behavior.

3 Discrepant drinkers are couples in which the husband drinks more than the wife in both frequency and quantity.

To capture more fully the complexity of the notion of a drinking partnership, Roberts and Leonard (in press) tried to identify different types of naturally occurring drinking partnerships in early marriage. To identify drinking-partnership profiles, patterns of six drinking and context variables were analyzed: the husband’s and wife’s drinking frequency, the husband’s and wife’s typical quantity consumed per drinking occasion, the percentage of the couple’s total drinking taking place in each other’s presence, and the percentage of the couple’s drinking occurring in the home. Five different types of partnerships were identified, and their relation to marital functioning, marriage-related drinking consequences, and general drinking consequences (e.g., legal or work problems) was examined (see table). Consistent with our conception of the influence of the drinking partnership, profiles characterized by high levels of individual consumption were not uniformly associated with lower marital quality. For example, couples in one profile scored high on the quality indices, even though both partners consumed alcohol in quantities above the mean and did so more frequently than partners in any other profile group. However, these couples consumed their alcohol in an “intimate” context, at home and together. In contrast, the two profiles associated with less optimal marital functioning were characterized by *discrepant* partner consumption patterns. In both of these profiles, the spouse who consumed more alcohol showed a pattern of higher quantity than frequency of consumption. The significant relationships between drinking partnership profiles and marital functioning held after statistically controlling for each partner’s average daily consumption. This result strongly suggests that the nature of a couple’s drinking partnership affects both husbands’ and wives’ marital quality independent of alcohol consumption.

## Summary

The empirical research suggests that the transition to marriage in early adulthood is associated with declines in alcohol consumption and alcohol-related problems. This transition also is associated with major structural and psychological changes at the individual, couple, and social-network levels. It is unclear, however, which of these changes are responsible for the “protective” effects of marriage on alcohol consumption. Furthermore, it is critical that research not uniformly view drinking as completely inimical to marriage. Clear risks to the relationship are associated with problem drinking. However, an assessment of the functional requirements of a marriage, along with an examination of how individual drinking patterns and, most critically, the drinking partnership affect those requirements, would further a thorough understanding of the effect of alcohol use on marriage.

## Footnotes

1 The term “heavy alcohol use” is not used consistently in the alcohol research literature, and the studies referred to in this article likewise define the concept in different ways. In this article, heavy alcohol use is defined as consumption of six or more drinks per occasion at least once per week.

2 The term “alcoholism” is used in this article to encompass all levels of problem alcohol use and does not refer to a particular diagnostic system.
